# Novel Primate Model of Serotonin Transporter Genetic Polymorphisms Associated with Gene Expression, Anxiety and Sensitivity to Antidepressants

**DOI:** 10.1038/npp.2016.41

**Published:** 2016-04-20

**Authors:** Andrea M Santangelo, Mitsuteru Ito, Yoshiro Shiba, Hannah F Clarke, Evelien HS Schut, Gemma Cockcroft, Anne C Ferguson-Smith, Angela C Roberts

**Affiliations:** 1 Department of Physiology, Development and Neuroscience, University of Cambridge, Cambridge, UK; 2 Behavioural and Clinical Neuroscience Institute, University of Cambridge, Cambridge, UK; 3 Department of Genetics, University of Cambridge, Cambridge, UK; 4 Brain Center Rudolf Magnus, Department of Translational Neuroscience, University Medical Center Utrecht, Utrecht, The Netherlands

## Abstract

Genetic polymorphisms in the repeat upstream region of the serotonin transporter gene (*SLC6A4*) are associated with individual differences in stress reactivity, vulnerability to affective disorders, and response to pharmacotherapy. However, the molecular, neurodevelopmental and psychopharmacological mechanisms underlying the link between *SLC6A4* polymorphisms and the emotionally vulnerable phenotype are not fully understood. Thus, using the marmoset monkey *Callithrix jacchus* we characterize here a new neurobiological model to help to address these questions. We first sequenced the marmoset *SLC6A4* promoter and identified a double nucleotide polymorphism (−2053AC/CT) and two single-nucleotide polymorphisms (−2022C/T and −1592G/C) within the repeat upstream region. We showed their association with gene expression using *in vivo* quantitative PCR and with affective behavior using a primate test of anxiety (human intruder test). The low-expressing haplotype (AC/C/G) was linked with high anxiety while the high-expressing one (CT/T/C) was associated with an active coping strategy in response to threat. Pharmacological challenge with an acute dose of the selective serotonin reuptake inhibitor, citalopram, revealed a genotype-dependent behavioral response. While individuals homozygous for the high anxiety-related haplotype AC/C/G exhibited a dose-dependent, anxiogenic response, individuals homozygous for the low anxiety-related haplotype CT/T/C showed an opposing, dose-dependent anxiolytic effect. These findings provide a novel genetic and behavioral primate model to study the molecular, neurodevelopmental, and psychopharmacological mechanisms that underlie genetic variation-associated complex behaviors, with specific implications for the understanding of normal and abnormal serotonin actions and the development of personalized pharmacological treatments for psychiatric disorders.

## Introduction

Serotonin regulates multiple physiological and behavioral processes crucial for emotional homeostasis and survival. Among these, the serotoninergic system orchestrates the response to threats and its adjustment to specific contexts. However, an exacerbated response to uncertain, but non-threatening, stimuli can lead to negative adaptive outcomes and constitutes one of the core features of anxious behavior (Grupe and Nitschke, 2013). Subjects suffering from affective disorders, including anxiety and depression, present alterations in serotonin-related functions. Of particular importance is the serotonin transporter, a key protein responsible for the reuptake of serotonin from the synaptic cleft and termination of its action (Kristensen *et al*, 2011).

Genetic polymorphisms in the serotonin transporter gene (*SLC6A4*) have long been associated with individual differences in reactivity to early-life stress and vulnerability to affective disorders in humans (Canli and Lesch, 2007; Caspi *et al*, 2010; Lesch *et al*, 1996). The most studied polymorphism is a variable number of tandem repeats (VNTR) within the upstream repeat region, with *short* alleles being linked to emotionally vulnerable phenotypes and reduced *SLC6A4* expression, both in humans and in macaques (Lesch *et al*, 1996; Spinelli *et al*, 2007). However, even after taking into account gene × environment interactions (Caspi *et al*, 2003, 2010) and a single-nucleotide polymorphism in the human long allele that confers a *short* allele phenotype (Hu *et al*, 2006), discrepancies exist among these gene–behavioral association studies (Munafò and Flint, 2004). Despite these discrepancies, *in vitro* expression analyses have shown that the *SLC6A4* repeat region regulates gene expression and that genetic variation at this locus has an impact on expression levels (Bennett *et al*, 2002; Heils *et al*, 1996).

In addition, correlational imaging studies have shown that even though there are no consistent genotype-dependent changes in serotonin transporter binding in the adult brain, there are chemical, structural, and functional alterations in neural circuits involved in emotional processing in *short* allele carriers (Jedema *et al*, 2010; Pacheco *et al*, 2009; Pezawas *et al*, 2005). Serotonin levels early in life are crucial for the development of the central nervous system and the emotional profile in adulthood (Suri *et al*, 2015). Thus, it has been proposed that the *short*-allele adult phenotype may be the result of changes in neural circuitry due to increased serotonin levels during neurodevelopment and the interaction between this emotionally vulnerable brain with stressful early life experiences (Ansorge *et al*, 2007).

More recently, large pharmacogenomic studies have shown an interaction between *SCL6A4* genotype and the efficacy of selective serotonin reuptake inhibitors (SSRIs) in the chronic treatment of anxiety and depression, with *short*-allele carriers presenting a lower remission rate (Keers *et al*, 2011; Serretti *et al*, 2007). It has also been theorized (Harmer and Cowen, 2013) that individual differences in subsequent treatment efficacy may be related to an initial anxiogenic effect seen during the early stages of SSRI treatment in various patient populations (Kent *et al*, 1998) and healthy volunteers (Bigos *et al*, 2008; Browning *et al*, 2007; Grillon *et al*, 2007). However, whether the acute anxiogenic effect of SSRIs is related to the *SCL6A4* VNTR remains unknown, with few studies having been performed in healthy volunteers (Hinkelmann *et al*, 2010).

Despite this increasing body of evidence strongly linking polymorphisms within the *SCL6A4* VNTR with genotype-specific gene regulation, neurodevelopmental changes, vulnerability to psychiatric disorders, and response to pharmacotherapy, our understanding of the underlying molecular, neurodevelopmental, and neuropsychopharmacological mechanisms is poor. Hence, it is crucial to characterize an animal model that will allow us to advance our understanding of these *SLC6A4* polymorphisms and vulnerable phenotypes associations, in order to develop effective psychopharmacological treatments. We characterize here such a novel genetic and behavioral model in the common marmoset *Callithrix jacchus*, a species that has gained increasing popularity in molecular neuroscience since the completion of the marmoset genome (Flicek *et al*, 2011) and the generation of the first transgenic marmoset (Sasaki *et al*, 2009). Moreover, given their small size, fast reproductive rate, short developmental period, and similarity of brain organization with that of humans, marmosets are becoming an ideal primate model to study the neurobiological mechanisms underlying affective disorders (Sawada *et al*, 2014).

In the present study, we characterize the entire marmoset *SLC6A4* repeat upstream region, identify novel sequence polymorphisms, and show their association with both gene expression levels and affective responses to threat. In addition, we reveal a variant-dependent behavioral response to threat after an acute dose of an SSRI, citalopram. Altogether, these findings establish a robust primate model for future molecular and developmental studies of the neurobiological mechanisms underlying individual differences in affective behavior, vulnerability to affective disorders, and pharmacotherapeutic efficacy.

## Materials and Methods

### Animals and Housing

Common marmosets *C. jacchus* (age: 29.9±1.2 months, weight: 433.8±9.1 g; see Supplementary Table S1 for sample size summary) were bred on site at the Innes Marmoset Colony (Behavioral and Clinical Neuroscience Institute). Genotyped animals were housed in pairs or in families. Animals included in the *SLC6A4* gene expression, behavioral, and pharmacological studies were only housed in pairs. Family relationship information among these animals can be found in Supplementary Figure S1. Temperature (24 °C) and humidity (55%) conditions were controlled and a dawn/dusk-like 12 h-period was maintained. They were provided with a balanced diet and water *ad libitum*. All procedures were performed in accordance with the project and personal licenses held by the authors under the UK Animals (Scientific Procedures) Act 1986.

### Cloning and Sequencing of the Marmoset SLC6A4 Repeat and Promoter Regions

A 2.4 kb-fragment spanning from −2.3 kb to the first exon of the *SLC6A4* marmoset gene was cloned using a PCR-based strategy. Briefly, the exon 1 and the proximal promoter region were amplified using the following primers: *SLC6A4*-AF, *SLC6A4*-AR, *SLC6A4*-BF1, *SLC6A4*-BR2, *SLC6A4*-CF1, *SLC6A4*-CF2, and *SLC6A4*-CR (Supplementary Table S2). HotStarTaq DNA Polymerase (Qiagen, UK) was used in a MJ Research PTC-200 thermal cycler (conditions: activation 16 min at 94 °C, 50 cycles of 30 s at 94 °C, 30 s at *T*_A1_ °C (*T*_A1_=from 54 to 60 °C, according to each primer Tm) and 1 min at 72 °C; and termination 5 min at 72 °C). The 300 bp upstream region of the promoter was cloned using inverse PCR methods with *NspI* and the following primers: IPCR-F1 and IPCR-R1 (conditions: activation 16 min at 94 °C, 30 cycles of 30 s at 94 °C, 30 s at *T*_A2_ °C (*T*_A2_= from 74 to 58 °C step-down program) and 4 min at 72 °C; and termination 5 min at 72 °C). The PCR products were cloned using the TOPO TA cloning kit system (Invitrogen Ltd, UK). Inserts were sequenced by GeneService Ltd (Cambridge) and consensus sequence was annotated in Nucleotide database (http://www.ncbi.nlm.nih.gov/nuccore) with the accession number HG515029 (Supplementary Figure S2).

### Determination of the SLC6A4 Transcription Start Site

Total RNA was extracted from marmoset Raphe nuclei by homogenization with TRI Reagent (Sigma) using the MagNA Lyser Instrument (Roche), followed by chloroform extraction and ethanol precipitation. RNA pellets were washed in 70% EtOH and resuspended in 50 μl of water and stored at −80 °C. To amplify the 5′ cDNA sequence of marmoset *SLC6A4*, a system for rapid amplification of cDNA ends (5′RACE) was used on total RNA extract using First ChoiceRLM-RACE kit (Ambion), following the manufacturer's protocol (see Supplementary Materials and Methods and Supplementary Table S2).

### Repeat Region Sequence Alignment

Marmoset repeats were characterized by multiple sequence alignments. *C. jacchus* gene sequences were obtained from our own *SLC6A4* clone sequencing and from the Ensembl database (www.ensembl.org). Human and other primate *SLC6A4* sequences (*Macaca mulatta* and *Pongo pygmaeus*) were obtained from Ensembl database. The *Gorilla gorilla* (accession number AB061805.1) and *Saguinus oedipus* (accession number AB326308.1) were obtained from the NCBI website (www.ncbi.nlm.nih.gov/nuccore). To determine the first and last repeats of the marmoset region, a preliminary alignment was performed with the human, macaque, and marmoset sequences using Clustal Omega online (http://www.ebi.ac.uk/Tools/msa/clustalo/) (Supplementary Figure S3). The internal repeats were aligned manually for all primate species based on the Lesch *et al* (1997) repeat consensus sequence unit (Supplementary Figure S4).

### Genotyping

Blood samples were taken from the femoral vein under sedation (0.1 ml i.m., Vetalar V 100 mg/ml; Pfizer, UK). A syringe was prefilled with ACD (acid–citrate–dextrose: 12.5 g/l Na citrate, 10 g/l D-glucose, 6.85 g/l citric acid) anticoagulant. Genomic DNA (gDNA) extraction was performed using Dneasy Blood & Tissue kit (Qiagen) (yield 2–6 μg per sample). Hair follicles were plucked from the animal's back. Samples were processed using the QIAamp DNA Micro kit for forensic casework samples (Qiagen) (yield 0.5–1.2 μg per sample). Primers were designed to flank the *SLC6A4* repeat region: RPRF and RPRR (Supplementary Table S2). HotStarTaq Plus DNA Polymerase (Qiagen) was used in a BioRad C1000 thermal cycler (conditions: activation 15 min at 94 °C; 44 cycles of 30 s at 94 °C, 30 s at 55 °C and 1 min at 72 °C; and termination 5 min at 72 °C). The PCR product was visualized in an agarose gel, purified using the Mini Elute PCR Purification Kit (Qiagen) and sent for sequencing (Source BioScience, Cambridge, UK). Primers used for sequencing can be found in Supplementary Table S2.

#### Chimaerism

When studying the behavioral and pharmacological effects, which are dependent upon the brain, animals were genotyped using hair follicles, which showed the lowest level of chimaerism (Benirschke *et al*, 1962; Sasaki *et al*, 2009), and the same genotype as the brain (Supplementary Table S3). When measuring RNA in blood, samples were genotyped using genomic DNA extracted from the same blood tissue, taking into account the chimaerism. Captive (Bethesa, USA) and free-range (Rio Grande do Norte, Brazil) populations of marmosets were genotyped using hair follicles (Supplementary Table S4).

### Expression Assay with qPCR

Total RNA was extracted using the QIAamp RNA Blood Mini kit (Qiagen). Samples were stored at −80 °C till use. cDNA synthesis and real time qPCR was performed using Brilliant II SYBR Green QRT-PCR Master Mix Kit, 1-Step (Agilent Technologies, UK) using primers spanning *SLC6A4* exons 12–13: *SLC6A4*-F and *SLC6A4*-R (Supplementary Table S2). Porphobilinogen deaminase gene (PBGD) was used as the reference gene using primers spanning exons 13–14: PBGD-F and PBGD-R. All primer combinations were designed to span exon–exon boundaries. All reactions were performed in duplicate for each individual and controls (conditions: cDNA synthesis 30 min at 50 °C, activation step 10 min at 95 °C, 40 two-step cycles of denaturation 30 s at 95 °C and combined annealing/extension 1 min at 60 °C, final melting curves to check specificity of the product) in a DNA Engine Opticon 2 thermocycler (MJ Research). Results were compared with a gene-specific standard curve and normalized to the expression of PBGD (van Lelyveld *et al*, 2008).

#### Data analysis

Results were analyzed with one-way ANOVA followed by LSD *post hoc* contrasts (SPSS Statistics 22.0). Data are presented as mean±SEM. A *p*<0.05 was considered statistically significant.

### Human Intruder Test

Anxious behavior was assessed using the human intruder test (HIT) (Agustín-Pavón *et al*, 2012). Marmosets were separated from their cage mate and restricted to the upper right-hand quadrant of their home cage (*separated phase*). After 8 min, an unfamiliar person entered the room. The intruder stood 40 cm from the front of the cage and stared at the marmoset, maintaining eye contact, for 2 min (*intruder phase*). Marmoset performance was recorded with a HD video camera (Genie CCTV-C5351/12, Korea) and a shotgun condenser microphone (Pulse-NPM702, Taiwan) with a preamplifier (Pulse-PLS00335, China). Once the intruder had left the room, recording went on for 5 min to observe the recovery of normal behavior. Several measures were scored off line by an experimenter blind to the genotype using the program JWatcher V1.0 (http://www.jwatcher.ucla.edu/): average distance (mean of the proportion of time spent in each of 15 locations with respect to the cage front); locomotion (proportion of time spent in translational movements between locations); jumps (number of jumps made to the front of the cage, towards the human intruder); bobbing event (number of rapid and repetitive side-to-side movements of the upper body while sitting and staring at the object of interest); and number of vocalizations (tsik, egg, tsik-egg and tse-like calls). For more details see Supplementary Materials and Methods and Supplementary Figure S5.

#### Data analysis

Distance and locomotion were scored and analyzed with repeated measures ANOVA for both separated and intruder phases. For the intruder phase, a principal component analysis (PCA) was performed on all eight variables, to extract the behavioral dimensions underlying the response to threat (Supplementary Table S5). The PC1 and PC2 derived from the PCA corresponded to Anxiety and Coping Strategy, respectively, based on the behavioral variable loadings (Agustín-Pavón *et al*, 2012). A two-way ANOVA was used to compare the two component scores (PC1 and PC2) between the *SLC6A4* genotypes followed by LSD pairwise comparisons. One-way ANOVA (or non-parametric test when normality was not achieved) followed by *post hoc* pairwise comparisons were also performed for each individual variable. All statistical analyses were performed with SPSS Statistics 22.0. Data are presented as mean±SEM. A *p*<0.05 was considered statistically significant.

### Pharmacological Manipulation on HIT

Twelve homozygous marmosets were included in this study (Supplementary Table S1). Animals were injected i.m. with citalopram (2.5 or 10 mg/kg) or vehicle (0.01 M PBS-HCl) 25 min before the intruder phase. We selected citalopram as it is a commonly used SSRI in the clinic and has been used when studying the impact of *SLC6A4* VNTR on treatment efficacy (Keers *et al*, 2011). HIT procedures were exactly the same as described above. To avoid habituation to the human intruder across sessions the intruder wore different realistic rubber human masks each session (Greyland Film spol. s r.o., Czech Republic). The experimental design was a latin square randomized by sex, genotype, and masks. Treatment order was the same for all individuals (lower dose, higher dose, and vehicle) with 2 weeks between each session.

#### Data analysis

To calculate the PCA scores for each treatment, the variable values were standardized using the mean and standard deviation of the control condition (injection with vehicle) of the experimental subpopulation used in this study (*N*=12). These standardized values were then used in a PCA function derived from the previously performed PCA that included the whole population (*N*=52). PCA scores and variables were analyzed using repeated measure ANOVA with one between subject factor (haplotype) and one within subject factor (treatment), using SPSS Statistics 22.0. Data are presented as mean±SEM. A *p*<0.05 was considered statistically significant.

## Results

### New Sequence Polymorphisms in the Marmoset *SLC6A4* Upstream Repeat Region are Linked to Gene Expression Levels

**Figure 1:**
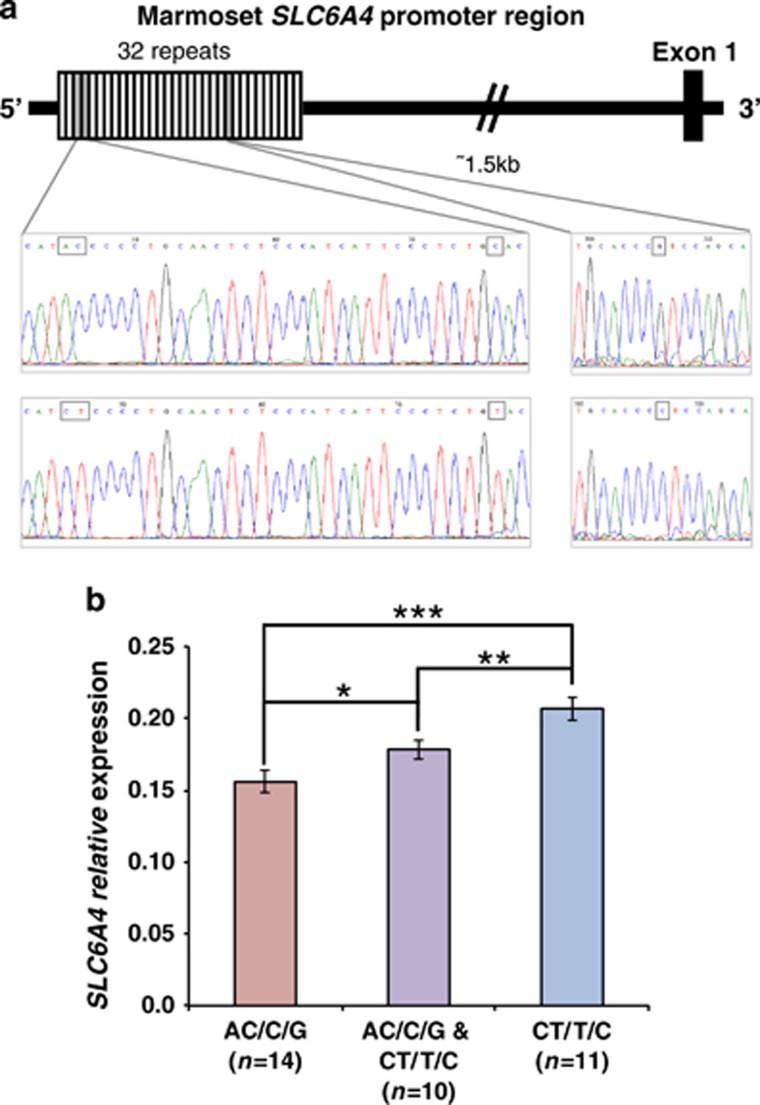
Genetic variation in the repeat upstream region of the marmoset *SLC6A4* gene and its association with gene expression levels. (a) Schematic representation of the marmoset *SLC6A4* promoter region showing 32 repeats. Third, fourth, and 23rd repeats containing the double and the two single-nucleotide polymorphisms, respectively, are shaded in grey. Representative examples of electropherograms of the most frequent haplotypes AC/C/G and CT/T/C are shown. (b) Relative expression values are shown (mean±SEM). One-way ANOVA *F*_(2,32)_=11.40, *p*<0.001 followed by *post hoc* LSD: AC/C/G *vs* CT/T/C *p*<0.001 (***), CT/T/C *vs* AC/C/G&CT/T/C *p*=0.020 (**), AC/C/G *vs* AC/C/G&CT/T/C *p*=0.047 (*).

To characterize genetic variation at the marmoset *SLC6A4* repeats, we cloned the promoter region from −2.3 kb to the first exon using a PCR-based method (Supplementary Table S2) and generated a consensus sequence (accession number HG515029) (Supplementary Figure S2). Using the 5′-RACE technique we experimentally identified the marmoset *SLC6A4* transcription start site (highlighted in Supplementary Figure S2) and determined the repeat boundaries by performing a series of sequence alignments, revealing the presence of 32 repeats (Supplementary Figures S3 and S4). We found no variation in the number of tandem repeats in our colony (*N*=144 animals). Instead, we identified sequence polymorphisms: one dinucleotide polymorphism in the third repeat (−2053AC/CT) and two single-nucleotide polymorphisms in the fourth (−2022C/T) and the 23rd (−1592G/C) repeats (Figure 1a). The haplotypes AC/C/G and CT/T/C showed high frequencies in our colony (49.6 and 42.4%, respectively) while the CT/C/G haplotype was less common (8%) and no other combination was detected (Supplementary Table S1). These haplotypes were also found in 62 animals from the marmoset colony maintained at the National Institute of Neurological Disorders and Stroke (Bethesda, United States) and the AC/C/G and CT/T/C haplotypes were also present in 47 individuals from free-ranging marmoset families living at the FLONA of Nísia Floresta field station—ICMBio (Rio Grande do Norte, Brazil). Genotypic frequencies of these captive and free-ranging populations are provided in Supplementary Table S4.

Owing to the low frequency of the CT/C/G haplotype and subsequent low number of CT/C/G carriers in our colony, we focused our subsequent analysis on the most frequent haplotypes, AC/C/G and CT/T/C, the genotypic frequencies of which followed Hardy–Weinberg equilibrium (*X*_(1)_^2^=3.06) (Supplementary Table S1). Quantitative PCR studies have shown that *SLC6A4* mRNA levels in human brain and lymphoblast cultures are similar (Gyawali *et al*, 2010) and more recent studies have proposed lymphocytes as an alternative peripheral model of central serotonin function (Marazziti *et al*, 2013). Thus, we assessed differential gene expression between *SLC6A4* genotypes using marmoset lymphocytes (*N*=35). Marmosets homozygous for the AC/C/G haplotype showed a significant reduction of 25% in *SLC6A4* gene expression compared with individuals homozygous for the CT/T/C variant, with samples from heterozygous marmosets expressing intermediate levels (Figure 1b). This effect was independent of sex or age (Supplementary Table S6).

### The Marmoset *SLC6A4* Polymorphisms are Associated with Anxiety and Coping Strategy in Response to Threat

**Figure 2:**
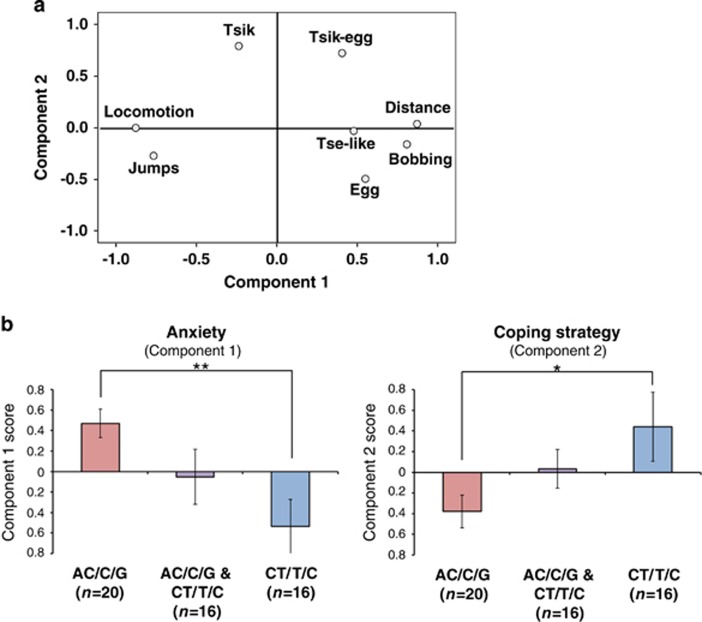
The marmoset *SLC6A4* polymorphisms are associated with individual differences in anxiety and coping strategy, assessed using the human intruder test (HIT). (a) Component plot in rotated space (variable loadings plot) illustrating the relationship of the individual behavioral measures with the two components derived from the principal component analysis (PCA). (b) Comparison of component behavioral scores (mean±SEM) derived from the PCA of the HIT performance. Left panel: Component 1 ‘Anxiety'. Right panel: Component 2 ‘Coping Strategy'. Two-way ANOVA, genotype × component interaction *F*_(2,49)_=9.36, *p*<0.001 (Power 97%), followed by *post hoc* LSD: AC/C/G *vs* CT/T/C, Component 1 *p*=0.002 (**), Component 2, *p*=0.014 (*).

To investigate the contribution of the marmoset *SLC6A4* polymorphisms to affective behavior, we used the HIT (*N*=52) (Agustín-Pavón *et al*, 2012). During the intruder phase, the marmosets spent more time at the back of the cage, maintaining some distance between them and the unfamiliar person (increased average distance) and moved around much less (reduced locomotion) compared with the separated phase (Table 1). The two principal components (PC1=anxiety and PC2=coping strategy) derived from the PCA explained together over 63% of the total variance (Figure 2a and Supplementary Table S5). When comparing individual PC scores between genotypes, marmosets homozygous for the low-expressing AC/C/G haplotype displayed significantly higher anxiety (higher PC1 scores, Figure 2b, left) and a more passive coping strategy (lower PC2 scores, Figure 2b, right) than the CT/T/C homozygotes, which showed the opposite behavioral pattern. This effect was independent of sex or age (Supplementary Table S6).

The increased PC1 scores in AC/C/G reflected reduced locomotion, increased distance from the intruder, and high numbers of head and body bobbing, and alarm calls (Figure 2a and Table 1), which are behaviors corresponding to high anxiety (Agustín-Pavón *et al*, 2012; Carey *et al*, 1992). In contrast, the increased PC2 scores in CT/T/C homozygous were related to high numbers of mobbing vocalizations implicated in active coping in response to stress (Bezerra and Souto, 2008; Cross and Rogers, 2006) Supplementary Videos S1 (for homozygous AC/C/G) and S2 (for homozygous CT/T/C) show representative examples of these two distinct behavioral phenotypes.

### The Marmoset *SLC6A4* Polymorphisms Modulate the Effect of an Acute Dose of a Serotonin Reuptake Inhibitor on the Response to Threat

**Figure 3:**
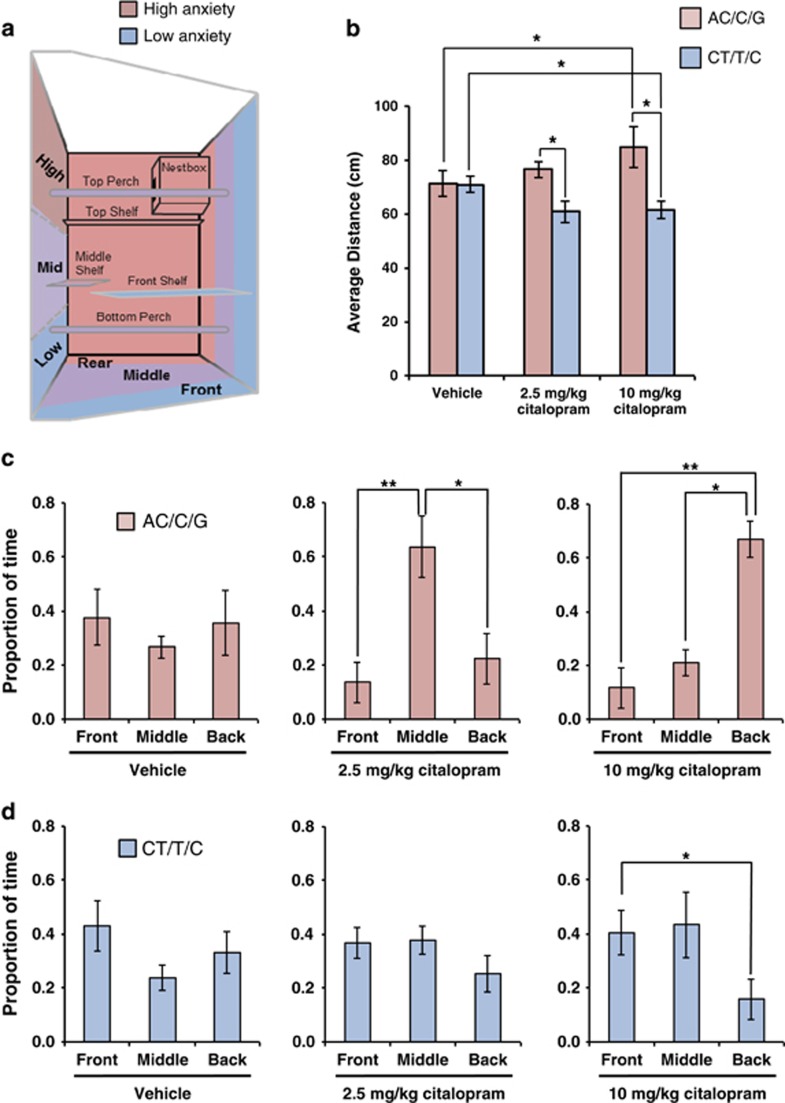
*SLC6A4* variant-specific anxiety response to an acute dose of a selective serotonin reuptake inhibitor, citalopram. Human intruder test (HIT) was used to assess anxiety levels in response to vehicle (V) and to a single dose of 2.5 mg/kg (D1) or 10 mg/kg (D2) citalopram, 25 min prior to the intruder phase. (a) Schematics of the home cage test quadrant in the HIT. High anxiety-related locations are shaded in red (high and rear) and low anxiety-related locations are shaded in blue (low and front). (b) Average distance. Repeated measures ANOVA, with within factor ‘treatment' and between factor ‘genotype'. Treatment × genotype interaction *F*_(2)_=4.214, *p*=0.030; followed by LSD pairwise comparisons. D1: AC/C/G *vs* CT/T/C *p*=0.017; D2: AC/C/G *vs* CT/T/C *p*=0.012; AC/C/G: V *vs* D2 *p*=0.012, and CT/T/C: V *vs* D2 *p*=0.031. (c, d) Proportion of time spent at different locations (mean±SEM): Front, middle and back, for each group of homozygous AC/C/G (c) and CT/T/C (d). Two-way repeated measures ANOVA with ‘location' (front, middle, back) and ‘treatment' (V, D1, D2) as within factors and ‘genotype' as between factor (AC/C/G, CT/T/C). Genotype × treatment × location interaction *F*_(4)_=6.530, *p*<0.001, followed by LSD *post hoc* comparisons. (c, middle panel) AC/C/G D1: front *vs* middle *p*=0.004; middle *vs* back *p*=0.027. (c, right panel) AC/C/G D2: front *vs* back *p*=0.001; middle vs back *p*=0.026. (d, right panel) CT/T/C D2: front vs back *p*=0.040. *p<0.05; **p<0.005.

To determine whether the marmoset *SLC6A4* polymorphisms influence the effect of SSRIs on affective responses, we compared HIT performance across homozygous genotypes after an acute dose of citalopram (*N*=12). Neither of the two acute doses of citalopram tested had any impact on the behaviors measured during the separated phase (Supplementary Table S7) nor on the PCA factor scores during the intruder phase (Supplementary Table S8), although PC1 scores were higher overall in the AC/C/G compared with the CT/T/C marmosets, replicating our previous finding in this subgroup. However, consistent with previous studies showing that distance from the human intruder is highly sensitive to anxiolytics (Carey *et al*, 1992), citalopram had a genotype-dependent effect specifically on average distance (Figure 3a and b). Specifically, there was a dose-dependent increase in average distance in high trait anxious AC/C/G marmosets, such that they spent more time away from the human intruder at the cage front, positioning themselves in the middle of the cage following the low dose (Figure 3c, middle panel) and at the back of the cage following the high dose (Figure 3c, right panel), indicative of heightened anxiety. In contrast, the CT/T/C homozygous marmosets exhibited the opposite behavioral pattern, moving closer to the intruder and thus reducing the average distance with increasing doses of citalopram, indicative of a reduction in anxiety (Figure 3d, right panel). This effect was independent of sex and age (Supplementary Table S6). Both genotypes showed reduced locomotion and numbers of jumps to the front in response to the acute citalopram (Supplementary Figure S6).

## Discussion

We report here, a new genetic and behavioral primate model highly relevant for the study of the underlying mechanisms of gene-affective behavior associations. We identify novel functional sequence polymorphisms within the marmoset *SCL6A4* upstream repeat region and show their association with individual differences in gene expression and negative affective behavior. In addition, we reveal a genotype-specific behavioral effect of an acute dose of the SSRI citalopram, with individuals carrying the low-expressing haplotype showing a dose-dependent anxiogenic response, in contrast to the anxiolytic response in those carrying the high-expressing haplotype.

Genetic variation in the upstream repeat region of the *SCL6A4* gene has been extensively investigated in both human and non-human primates (Inoue-Murayama *et al*, 2000; Lesch *et al*, 1997). In particular, a recent study in the marmoset showed no variation in the number of repeats; however, their sequence was incomplete (Pascale *et al*, 2012). Here, we have determined the full length of the marmoset repeat region with a total number of 32 repeats. Instead of a VNTR, we have identified three sequence polymorphisms within the repeat region that parallel the behavioral and gene expression effects associated with the human and macaque VNTRs. The importance of sequence variation in this region is highlighted by the presence of a single-nucleotide polymorphism within the human repeat polymorphic region that is also linked to *SCL6A4* expression and psychiatric disorders (Hu *et al*, 2006). Moreover, the marmoset sequence polymorphisms reported here showed haplotype-specific gene expression levels that correspond with the expression changes associated with repeat length and sequence polymorphisms using human lymphocyte cell lines (Bennett *et al*, 2002; Hu *et al*, 2006; Lesch *et al*, 1996).

Evidence that the haplotypes AC/C/G, CT/T/C and CT/C/G are not restricted to our colony is provided by their detection within the marmoset-breeding colony at the National Institute of Neurological Disorders and Stroke. The former two haplotypes are also present in five free-ranging marmoset families living at the FLONA of Nísia Floresta field station—ICMBio. The failure to detect the low frequent CT/C/G haplotype suggests that it may not be present in wild populations.

Consistent with reports in humans and macaques that have linked the *short* alleles with emotionally vulnerable phenotypes and anxiety traits, the low-expressing haplotype in the marmoset (AC/C/G) was also associated with high anxiety. However, this contrasts with a study in macaques which detected no association between the *SLC6A4* VNTR and ‘anxious temperament' using a non-eye contact HIT (Oler *et al*, 2010), although differential neural circuitry have been reported (Kalin *et al*, 2008). One possible explanation may be that the macaque and marmoset behavioral models are characterizing distinct trait anxiety phenotypes (Shackman *et al*, 2013; Shiba *et al*, 2014) that are differentially sensitive to *SLC6A4* VNTR. This is particularly likely since these primate models measure different behaviors in response to the human intruder in two quite distinct contexts, in the home cage in case of the marmoset and in a separate isolated room in macaques.

In this study we have also revealed an effect of the *SCL6A4* polymorphism on a second dimension of the affective response, namely coping strategy. The high-expressing haplotype CT/T/C was associated with an active coping strategy in response to threat. Although a role for *SLC6A4* variation in coping strategy has not been previously reported, differences in passive and active coping styles have been related to altered serotonin release in the dorsal raphe nucleus in response to stress (Amat *et al*, 2005). Moreover, an active coping style has been associated with increased aggressive behavior and reduced brain serotonin levels (Koolhaas *et al*, 2010).

An important question arising from these findings is why a gene–behavioral association was revealed in the marmoset, apparently, relatively easily compared with the mixed findings in humans? The answer may lie in the more complex phenotype of human, compared with marmoset, emotional behavior that is influenced not only by environmental and genetic factors but also a rich life experience. The contribution of genetic variation to individual differences in human behavior is thus attenuated by these other influences as well as by compensatory and homeostatic mechanisms. This can be offset, however, by studying such behavior in a non-human primate species where the emotional behavior is a less complex phenotype and the enormous variation in environment and life experiences between individuals can be dramatically reduced by studying a purpose-bred primate colony in a controlled environmental setting, as described here. Thus, we would argue for these reasons the effects of genotype–behavior relationships have been more easily revealed. In addition, when studying emotional behavior it is also important to recognize the variable array of responses that are displayed by individual animals to threat. Accordingly, when characterizing the repertoire of behaviors that marmosets display in response to a human intruder we took into account the ethological behavior of a marmoset when facing predators in the natural environment (Ferrari and Ferrari, 1990; Stevenson and Poole, 1976) and under experimental conditions (Barros, 2002; Cilia and Piper, 1997). Moreover, by using PCA, we were able to reveal the genotype-specific, haplotype dose-dependent effect of *SLC6A4* genetic variation on two dimensions of the threat response in the marmoset, emotionality and coping strategy.

Nonetheless, the putative contribution of each marmoset polymorphism to gene expression and behavior needs to be further investigated using, for example, *in vitro* controlled studies in marmoset cell culture (Shimada *et al*, 2012). In addition, the promoter region proximal to the transcription start site should also be explored for genetic variation possibly contributing to the phenotypes described here. Finally, we cannot rule out the possibility that a different genetic locus, co-segregating with the marmoset *SLC6A4* polymorphisms, may be also contributing to the genotype–phenotype associations. The development of transgenic and genome editing technologies will enable us to confirm these genetic–behavior associations in this primate model.

In addition to the association with distinct behavioral traits, we showed that the marmoset *SLC6A4* polymorphisms were also associated with individual differences in response to an SSRI. The AC/C/G high-trait anxious marmosets showed a dose-dependent anxiogenic response, as measured by average distance from the anxiety-provoking human. This result is of particular interest given a recent report showing increased fear reactivity after short-term citalopram administration in individuals high in neuroticism (Di Simplicio *et al*, 2014), an anxiety trait dimension associated with the *SLC6A4* VNTR in humans (Lesch *et al*, 1996). Contrary to this anxiogenic effect, the low-trait anxious CT/T/C marmosets exhibited a reduction in anxiety, that is, reduced distance from the anxiety-provoking human, in response to the acute high dose of citalopram. This latter effect is consistent with the early changes in cognitive and neurobiological processing of emotional stimuli detected after short-term SSRI administration (Harmer *et al*, 2006; Murphy *et al*, 2009) that may account for the later improvement of the clinical symptoms observed in *long* allele carriers.

**Figure 4:**
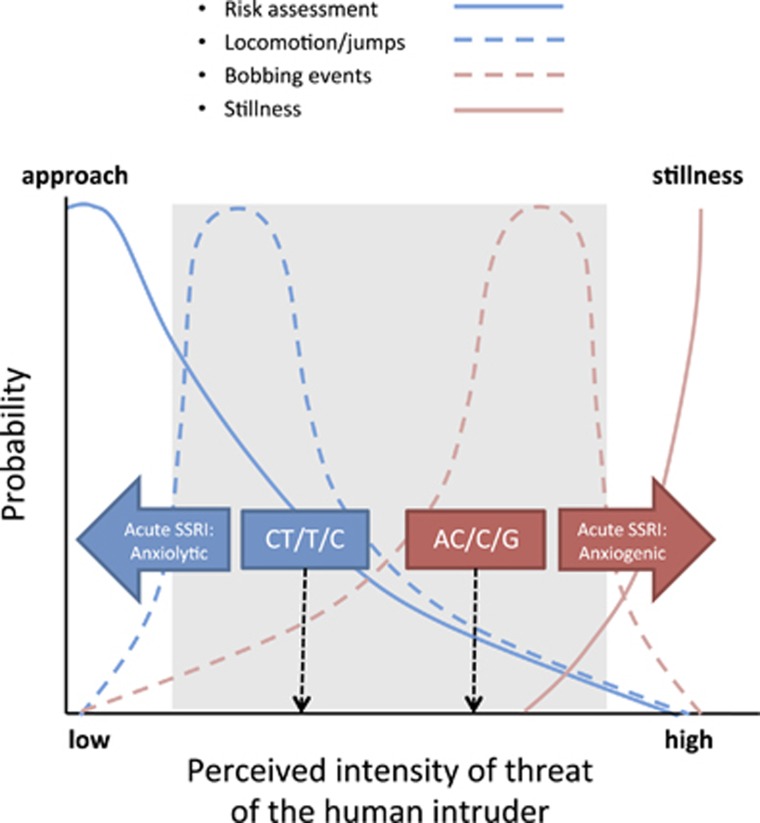
Schematic depicting the response to threat during the human intruder anxiety test. The graph indicates the probability of engaging in different behaviors in relation to the perceived intensity of threat. The normal range population described by our principal component analysis is shaded in gray. The AC/C/G group perceive the human intruder as a relatively high threat, showing high numbers of bobbing events, reduced locomotion and jumps, and primarily avoidance of the threat (reduced risk assessment). The CT/T/C group perceive the human intruder as a lower threat, showing fewer numbers of bobbing events, increased locomotion and jumps, and approach behavior to the threat (increased risk assessment/approach). Acute selective serotonin reuptake inhibitor (SSRI) in the AC/C/G group produces an anxiogenic effect with avoidance of the threat and further reduction in locomotion, jumps, and numbers of bobbing events, leading to an anxious state of stillness. In contrast, acute SSRI in the CT/T/C group induces an anxiolytic effect leading to increased approach behavior (increased inspection/risk assessment of the human intruder), with concomitant reduction in locomotion and jumps.

Although acute SSRI treatment produced genotype-dependent opposing effects on approach-avoidance behavior, its effects on other threat-related measurements were in the same direction, that is, reduction in both locomotion and jumps to the front. However, a reduction in the latter behaviors can be brought about by both increases and decreases in anxiety. The type of defense response adopted by an animal depends upon the result of the risk assessment performed, which takes into account the likelihood and proximity of the threat to determine whether to avoid immediately or to approach and gather more information (Blanchard *et al*, 2011; McNaughton and Corr, 2004). A high-threat risk assessment of the stimulus results in immediate avoidance behavior. In the current study, high anxious AC/C/G animals in response to an acute SSRI spent more time at the back of the cage (reduced risk assessment) and stayed very still, as reflected by reduced locomotion and jumps to the front (Figure 4). On the contrary, a low-threat risk assessment results in approach behavior and increased attention. This was the pattern of behavior observed in the low trait anxious CT/T/C group in response to acute SSRI, resulting incidentally in reduced locomotion and jumps to the front as they remained at the front for a large percentage of the time attending to the intruder.

Together, these findings bridge the gap between the finding of reduced responsivity to chronic SSRI treatment in *short*-allele carriers with anxiety disorders (Perna *et al*, 2005) and depression (Keers *et al*, 2011; Porcelli *et al*, 2012) and the individual differences in sensitivity to the anxiogenic effect of acute SSRIs (Harmer and Cowen, 2013). Currently, there are no studies that have specifically considered the relationship between the acute behavioral effects of SSRIs and *SLC6A4* genotypes with respect to treatment efficacy in patients with mood and anxiety disorders. This is an important issue though, since it is clinical practice to initiate SSRI treatment below therapeutic doses if anxiety symptoms are elicited in the early stages of treatment. By demonstrating genome-dependent differential effects of the *SLC6A4* polymorphism on the behavioral effects of acute SSRIs in marmosets, the present study provides support for the proposal that individual differences in the acute SSRI-induced anxiogenic effect may predict subsequent treatment efficacy (Harmer and Cowen, 2013).

In conclusion, our findings link genetic functional polymorphisms to differential *SLC6A4* expression and individual differences in complex affective primate behaviors, including sensitivity to pharmacotherapies. This new primate genetic model provides a unique tool for future investigations into the neurodevelopmental changes and physiological endophenotypes associated with serotonin genetic variation that leads to emotional vulnerability. Importantly, it also provides a model to study the neurochemical and neurobiological mechanisms of SSRI actions and their interactions with serotonin genetic variation. Finally, with the recent development of transgenic (Sasaki *et al*, 2009) and stem cell (Shimada *et al*, 2012) biotechnologies in marmosets, it will be possible in the future to use this model to further characterize the molecular mechanisms regulating the serotoninergic system and develop more efficient and specific molecular therapies for the treatments of mood and affective disorders.

## Funding and disclosure

This work was supported by an MRC Programme (ACR;G0901884) and performed within the Behavioural and Clinical Neuroscience Institute, University of Cambridge, funded jointly by the Wellcome Trust and MRC. AMS was supported by a McDonnell Foundation grant (PIs: EPhelps, TW Robbins; co-investigators: ACR and JLeDoux; 22002015501) and currently supported by MRC; YS supported by the Long Term Student Support Program provided by Osaka University and the Ministry of Education, Culture, Sports, Science and Technology of Japan; HFC supported by MRC Career Development Award and ACFS/MI supported by grants from the MRC and Wellcome Trust. GC supported by the Behavioural and Clinical Neuroscience Institute, Cambridge, UK. EHSS was self-funded. The authors declare no conflict of interest.

## Figures and Tables

**Table 1:** Human Intruder Test Performance Summary

	***SLC6A4*** **genotypes**		
	**AC/C/G homozygous**	**AC/C/G and CT/T/C heterozygous**	**CT/T/C homozygous**
*Separated phase*			
Locomotion (s)^a^	10.04±1.75	13.44±2.45	17.66±2.53
Distance (cm)^b^	71.00±4.06	68.00±4.45	64.34±4.62
			
*Intruder phase*			
Locomotion (s)^a^	5.88±0.91^c^	8.17±2.05	13.27±2.43
Distance (cm)^b^	87.75±2.56	81.57±4.98	75.13±4.46
Bobbing	54.00±5.43^d^	31.00±5.80	25.37±5.20
Jumps	0.90±0.25	1.44±0.62	2.31±0.72
Egg calls	21.55±3.31^e^	11.87±2.95	8.94±2.46
Tse-like calls	8.35±2.62	12.37±±4.70	4.31±1.04
Tsik calls	0.65±0.36^f^	3.67±1.54	6.87±2.59
Tsik-Egg calls	13.35±3.69	10.94±3.78	17.50±6.00

Mean±SEM for each variable during separated and human intruder phases.

a Repeated measures ANOVA, square root transformed, separated *vs* test phases, significant main effect *F*_(1,49)_=10.10, *p*=0.003.

b Repeated measures ANOVA, square transformed, separated *vs* test phases, significant main effect *F*_(1,49)_=36.41, *p*=0.000, *p*<0.001.

c ANOVA, square root transformed, *F*_(2,49)_=4.20, *p*=0.021 followed by *post hoc* LSD: AC/C/G *vs* CT/T/C *p*=0.008, heterozygous *vs* CT/T/C *p*=0.036.

d ANOVA, *F*_(2,49)_=8.03, *p*=0.001 followed by *post hoc* LSD: AC/C/G *vs* heterozygous *p*=0.004, AC/C/G *vs* CT/T/C *p*=0.001.

e ANOVA, square root transformed, *F*_(2,49)_=5.95, *p*=0.005 followed by *post hoc* LSD: AC/C/G *vs* heterozygous *p*=0.013, AC/C/G *vs* CT/T/C *p*=0.002.

f Kruskal–Wallis *X*_2_^(2)^=10.46, *p*=0.005 followed by Mann–Whitney AC/C/G *vs* CT/T/C (sig. two-tailed) *p*=0.001.
