# Supplementary material for: Novel Primate Model of Serotonin Transporter Genetic Polymorphisms Associated with Gene Expression, Anxiety and Sensitivity to Antidepressants
Source: Neuropsychopharmacology. 2016 Apr 20;41(9):2366–76. doi: 10.1038/npp.2016.41 (PMC4946067; doi:10.1038/npp.2016.41)
Supplement: Supplementary Information [file npp201641x1.pdf]

Supplementary Information

Title: Novel primate model of serotonin transporter genetic polymorphisms associated with gene expression, anxiety and sensitivity to antidepressants

Running title: New serotonin transporter polymorphism in marmoset

Andrea M. Santangelo, PhD[1,2], Mitsuteru Ito, PhD[3], Yoshiro Shiba, PhD[1], Hannah F. Clarke, PhD[1,2], Evelien H.S. Schut, MSc[4], Gemma Cockcroft[1,2], Anne C. Ferguson-Smith, Prof[3*] and Angela C. Roberts, Prof[1,2*]

[*]Joint senior authors

[1]Department of Physiology, Development and Neuroscience, University of Cambridge, Downing Street, CB2 3DY, Cambridge, UK

[2]Behavioural and Clinical Neuroscience Institute, University of Cambridge, Downing Street, CB2 3EB, Cambridge, UK

[3]Department of Genetics, University of Cambridge, Downing Street, CB2 3EH, Cambridge, UK

[4] Brain Center Rudolf Magnus, Department of Translational Neuroscience, University Medical Center Utrecht, Utrecht, The Netherlands.

Correspondence information: Andrea M. Santangelo, PhD, Department of Physiology, Development and Neuroscience, University of Cambridge, Downing Street, CB2 3DY, Cambridge, UK.

Tel: +44 1223 339 012, Fax: +44 1223 339 014, Email: as966@cam.ac.uk

Supplementary Materials and Methods

Rapid amplification of cDNA ends (5'RACE)

Nested PCR was performed using nested forward primers corresponding to the 5' RACE Adapter sequence (provided by the kit) and pair combinations of nested reverse gene-specific primers (GSP) for *SLC6A4*, designed based on alignment with the human promoter, using the Primer3 (http://primer3.ut.ee/) (**Table S2**). PCR assays contained (outer/inner) 1µl of cDNA/outer PCR product, 1XPCR buffer, 0.2mM dNTPs, 0.4µM 5'RACE Outer/Inner primer, 0.4µM GSP, 0.6u HotStarTaq plus (QIAGEN) made up to a total volume of 25µl with high purity water. PCR cycling conditions (touchdown PCR): 5 min at 96°C, 30 sec at 94°C, 30 sec at 72°C and 2 min at 72°C. The annealing temperature was reduced 2°C every four cycles and then to 54°C for 30 cycles with a final extension of 10 min at 54°C. PCR products from the 5'RLM-RACE nested PCR were extracted from gel and purified (MinElute Gel Extraction Kit,QIAGEN). Gel-extracted PCR products were cloned into the PGEM-T EasyVector (Promega) according to the manufacturer's instructions. Positive DNA clones were sent to Source BioScience for DNA sequencing.

Variables scored in the Human Intruder Test (HIT)

Average distance. The proportion of time spent in each of fifteen locations (n) (**Fig. S5A** and **B**) was scored. An average distance (cm) was calculated using the following formula:

Average distance= [Mean distance of location (n) x proportion of time spent in location (n)] summed across all 15 locations.

'Mean distance of location' is the hypotenuse from the cage front floor to the middle point of each location (table at **Fig. S5C**).

Locomotion. Proportion of time spent in translational movements between locations (self-propulsion involving all four limbs).

Head and body bobbing. Number of rapid and repetitive side-to-side movements of the upper body while sitting and staring at the object of interest (Barros, 2002).

Jumps. Number of jumps made to the front of the cage, towards the human intruder.

Number of *vocalizations*. *Egg calls*, associated with vigilant behavior (Bezerra and Souto, 2008). *Tsik calls*, uttered alone or in series, primarily mobbing calls (Cross and Rogers, 2006). *Tsik-egg calls*, usually one tsik followed by 1-3 eggs (Shiba *et al*, 2015). *Tse calls*, similar but distinguishable from tsik calls, emitted in less threatening situations. (Tse and Tse-egg calls were highly correlated with one another so they were combined for purposes of analysis into Tse-like calls). The vocalizations were characterized using their sonograms (**Fig. S5D**).

Vocalizations and *head and body bobbing* behavior were observed only in the presence of the human intruder.

For examples of the behaviors described above see **Video S1** and **Video S2**.

Table S1| Sample sizes				
Study:	Population Genotypes	SLC6A4 Expression	Anxiety Phenotype	Response to citalopram
Homozygotes				
AC/C/G	42 (17,25)	14 (5,9)	20 (10,10)	5 (2,3)
CT/T/C	32 (15,17)	11 (7,4)	16 (8,8)	7 (4,3)
CT/C/G	3 (1,2)	0	0	
Heterozygotes				
AC/C/G&CT/T/C	50 (28,22)	10 (4,6)	16 (8,8)	
AC/C/G&CT/C/G	9 (5,4)	0	0	
CT/T/C&CT/C/G	8 (4,4)	0	0	
Total	144	35	52	12

Table S1| Numbers of animals genotyped from our colony at the time (*Population Genotypes* column), numbers used in real time PCR assays (*SLC6A4 Expression* column), numbers in Human Intruder Test to measure anxiety phenotype (*Anxiety phenotype* column), and in the pharmacological study to measure anxiety in response to citalopram (*Response to citalopram* column). Numbers in () represent females and males, respectively.

Table S2| Primer sequences		
**Name**	**Forward/reverse**	**Sequence (5'→3')**
SLC6A4 promoter region: PCR-based cloning strategy		
SLC6A4-AF	Forward	CGCGGGGAAGCATTAAGT
SLC6A4-AR	Reverse	AGGCGGGGAAGAAGGTCT
SLC6A4-BF1	Forward	AGGTGTCCGAGGTCAAGA
SLC6A4-BR2	Reverse	GAATTTTTGCGTCACTTTGAG
SLC6A4-CF1	Forward	CAACTCCCTGTACCCCTCCT
SLC6A4-CF2	Forward	CCTAGGATCGCTCCTGCAT
SLC6A4-CR	Reverse	GCGTGTAGGGTTCTGCTTGT
IPCR-F1	Forward	ACCGGCTGCACAGTTTTC
IPCR-R1	Reverse	TATAGAGGGACGGAGCTGGA
SLC6A4 TSS: 5'RACE method		
5RACE5R	Reverse	GGCTGGGGAGGTGGTCAGG
5RACE6R	Reverse	GGGCTGGGGAGGTGGTCA
5RACE7R	Reverse	ACTGCCAGGCGGAGGCTGGT
SLC6A4 repeat region: genotyping		
RPRF	Forward	CAGACAACCGTGTTCATCTG
RPRR	Reverse	GATTCTAGTGCCACCTAGAC
SeqF1	Forward	AGCAGCACCTAACCCTCCTA
SeqF2	Forward	TCCCCACTAGGCATTGCTAC
SLC6A4 gene expression: real time PCR		
SLC6A4-F	Forward	GTTCTACGGCATCACTCAGTTC
SLC6A4-R	Reverse	GCTGATGGCCACCCAGCAGATC
PBGD-F	Forward	AGGATGGGCAACTGTACCTGAC
PBGD-R	Reverse	ATGGATTGTAGCCTGCATGGTG

Table S2| Primers *SLC6A4*-AF, *SLC6A4*-AR, *SLC6A4*-BF1, *SLC6A4*-BR2, *SLC6A4*-CF1, *SLC6A4*-CF2 and *SLC6A4*-CR were used to amplify three regions of the marmoset promoter (region A closer to Exon 1, B middle region and C further upstream exon 1). Primers IPCR-F1 and IPCR-R1 were used in inverse PCR to amplify the 5' end of the repeat region. Primers 5RACE5R, 5RACE6R and 5RACE7R were used as gene-specific, nested, reverse primers in the 5'RACE methods. Primers RPRF and RPRR were used for genotyping. Primers SeqF and SeqR were used to sequence the polymorphic region for genotyping. Primers *SLC6A4*-F and *SLC6A4*-R were used to amplify *SLC6A4* RNA (gene of interest) and primers PBGD-F and PBGD-R to amplify PBGD RNA (reference gene) in real time PCR assays to assess *SLC6A4* expression.

**Table S3| Genotyping results using gDNA from hair follicles and brain**	
	N
Homozygotes	
AC/C/G	4 (2,2)
CT/T/C	5 (2,3)
CT/C/G	0
Heterozygotes	
AC/C/G&CT/T/C	12 (5,7)
AC/C/G&CT/C/G	1 (1,0)
CT/T/C&CT/C/G	4 (1,3)
Total	26

Table S3| Numbers of animals genotyped using genomic DNA from both hair follicles and brain tissue (brainstem). Numbers in () represent females and males, respectively.

Table S4| Genotypic frequencies of captive and free-range populations		
Genotype	Captive (Bethesda, US)	Free-range (Rio Grande do Norte, Brazil)
AC/C/G homozygous	36 (13,23)	6 (2, 4)
CT/T/C homozygous	0	21 (12, 9)
CT/C/G homozygous	0	0
AC/C/G & CT/T/C	11 (7,4)	20 (11, 9)
AC/C/G & CT/C/G	13 (4,9)	0
CT/T/C & CT/C/G	2 (2,0)	0
Total	62	47

Table S4| Genotypic frequencies from captive marmoset at the colony maintained at the National Institute of Neurological Disorders and Stroke (Bethesda, United States) and free-ranging marmoset families living at FLONA of Nísia Floresta field station - ICMBio (Rio Grande do Norte, Brazil). Numbers in () represent females and males, respectively.

Table S5| Principal component analysis. Rotated Factor Loadings

Measurements	Component 1	Component 2
Locomotion	**-0.88**	-0.01
Distance	**0.88**	0.03
Bobbing	**0.81**	-0.16
Jumps	**-0.76**	-0.28
Egg calls	**0.55**	**-0.50**
Tse-like calls	**0.48**	-0.02
Tsik calls	-0.24	**0.79**
Tsik-Egg calls	0.41	**0.72**
Eigenvalues	3.57	1.48
% of variance	44.61	18.54

Note: Factors loading over 0.44 (20% contribution) appear in bold.

Table S5| Principal Component Analysis (PCA) output for human intruder test. PCA was conducted on the 8 variables with orthogonal rotation from all 52 marmosets. The Kaiser-Meyer-Olkin measure verified the sample adequacy for the analysis, KMO=0.73 and all KMO values for individual items were ≥0.5. Bartlett's test of sphericity $X^2_{(28)}$=170.3, p<.001 indicated that correlations between variables were sufficiently large for PCA. An initial analysis was run to obtain the eigenvalues for each component in the data. Two components had eigenvalues above Kaiser's criterion of 1 and in combination explained 63.15% of the variance. The behaviors and vocalizations that cluster on each component suggest that component 1 (PC1) represents Anxiety and component 2 (PC2), Coping Strategy.

**Table S6| Analysis of the effect of sex and age on *SLC6A4* gene expression, behavioral performance in the human intruder test (HIT) and anxiety response to citalopram**			
**Variable**	**Analysis**	**F**	***p***
***SLC6A4* expression**	One-way ANOVA sex	$F_{(1,33)}$=2.78	.105
	Two-way ANOVA haplotype x sex	$F_{(2,29)}$=2.12	.138
	One-way ANOVA haplotype, sex as covariate	$F_{(1,31)}$=.945	.338
	One-way ANOVA haplotype, age as covariate	$F_{(1,31)}$=3.94	.056[a]
**PC1 and PC2**	Two-way ANOVA PC x sex	$F_{(1,50)}$=1.83	.182
	Three-way ANOVA PC x haplotype x sex	$F_{(2,46)}$=.161	.852
	Two-way ANOVA PC x haplotype, sex as covariate	$F_{(1,48)}$=2.46	.123
	Two-way ANOVA PC x haplotype, age as covariate	$F_{(1,48)}$=.075	.786
**Average distance**	Repeated measures treatment x sex	$F_{(2,20)}$=1.49	.249
	Repeated measures treatment x haplotype x sex	$F_{(2,16)}$=.367	.699
	Repeated measures treatment x haplotype, sex as covariate	$F_{(2,18)}$=1.97	.168
	Repeated measures treatment x haplotype, age as covariate	$F_{(2,18)}$=.738	.492

[a] One-way ANOVA haplotype, age as covariate, haplotype main effect $F_{(2,31)}$=7.91, *p*=.002

Table S6| Analysis of the effect of sex and age on the variables *SLC6A4* relative gene expression, PC1 and PC2 in the human intruder test (HIT) and average distance in response to an acute dose of citalopram. First, sex has been analyzed as main factor, then as a second factor interacting with haplotype and finally added as a covariate. Age has been added as a covariate.

Table S7| Behavioral performance during human intruder test *separated phase* after a single dose of citalopram

	Distance (cm)			Locomotion (sec)			Jumps to the front		
	**Vehicle**	**2.5 mg/kg**	**10 mg/kg**	**Vehicle**	**2.5 mg/kg**	**10 mg/kg**	**Vehicle**	**2.5 mg/kg**	**10 mg/kg**
**AC/C/G**	66.4±3.2	71.4±5.3	67.2±5.5	4.1±1.0	3.2±0.7	1.7±0.5	2.6±0.5	1.2±0.6	1±0.5
**CT/T/C**	60.4±4.0	52.4±7.1	63.7±5.8	7.0±1.5	4.3±0.9	5.6±1.8	3.3±1.1	1.9±0.5	2.3±0.6

Table S7| Behavioral performance during control *separated phase* in the human intruder test after a single dose of 2.5 or 10 mg/kg of citalopram, 25 minutes prior the *intruder phase.* No significant effects were detected using repeated Measures ANOVA, with within subject factor 'treatment' and between subject factor 'genotype'. Mean±s.e.m.

Table S8| Human intruder PCA factor scores after single dose of citalopram						
	**PC1: Anxiety**			**PC2: Coping strategy**		
	**Vehicle**	**2.5 mg/kg**	**10 mg/kg**	**Vehicle**	**2.5 mg/kg**	**10 mg/kg**
**AC/C/G**	0.41±0.23	0.44±0.06	0.72±0.18	-0.38±0.44	-0.57±0.30	-0.29±0.29
**CT/T/C**	-0.30±0.21	-0.47±0.19	-0.22±0.12	0.27±0.19	0.57±0.33	0.43±0.31

Table S8| Anxiety response to selective serotonin reuptake inhibitor, citalopram. Human Intruder Test was used to assess anxiety levels in response to vehicle and to a single dose of 2.5 or 10 mg/kg of citalopram, 25 minutes prior the *intruder phase*. The component factor scores were calculated for each individual using standardization to the mean and SD of the vehicle, control condition (see Methods and Materials for details) and compared between genotypes using Repeated Measures ANOVA, with within factor 'treatment' and between factor 'genotype'. There was no significant interaction between treatment and genotype. Between subject effect Genotype: PC1: $F_{(1)}$=37.737 p<.0001, PC2: $F_{(1)}$=4.569 p<.058. Mean±s.e.m.

Figure S1:

(See file attached as Figure S1)

Figure S1| Family tree showing the relationship between the 52 marmosets included in the behavioral association study using the human intruder test of anxiety, which are indicated with a black border. The 12 marmosets used in the pharmacological study with citalopram are a subgroup of this cohort. Known genotype is indicated with a color code.

Figure S2:

```
-2.3 kb
CATGTTCACTTAAGTGGAACATTGGAGGCAGCAGACAACCGTGTTCATCTGAAAGGAGGAGCCCCCACCCCCATGCAGTCGCTGCCCCCAGAAT
AAAATTCCCAAGCTTGTTGGGGAATCCCCACTAGGCATTGCTACTCTGAATGCCAGCAGCACCTAACCCTCCTAACGT
1)      CCCTGCTGCACTCTTCCCTTCA
2)      CCCCCCACCCCCCGCACCCTCCCAGCAT
3)      ACCCCCTGCAACTCTCCCATCAT
4)      TCCCTCTGCACCCCTCCCAGCAT
5)      TCCCCCTGCACCCTCCCAGCAT
6)      CCCTCCTCCCCTCTGCACCCCTCCCAGCAT
7)      TCGCCCTGCACCCTCCCAGCAT
8)      CCCCCTTCACCCCTCCCTTCGT
9)      CCCCCCTGCATCCTCCCAGCAT
10)     CCCCCTGCACCCCTCCCAGCAT
11)     CCCCCCTGCATCCTCCCA
12)     CCCCTCTGCACCCCTCCCAGCAT
13)     CCCCCCTGCACCCTCCCAGCAA
14)     CCTCCCTCCCCCCGGAACCCCTCCCAGCAT
15)     TCCCCTGCACCCCTCCAGCAT
16)     CTCCCTTGCCCCAGACGT
17)     CGCCCCTGCACCCCTCCAGCAT
18)     TCCCCTTGTGCCCCCCAGAAT
19)     CTCCCTTATACCTCTCCCAGCAT
20)     CCCCCCCGTAACCCCTCCCAGCAT
21)     CCCCCTGCACCCCTCCAGCAT
22)     CTCCCTTGCCCCTAGACGT
23)     CACCCCTGCACCCGTCCAGCAT
24)     TCCCCTTGTGCCCCCCAGATC
25)     TCCCCTATACCTCTCCCAGCAT
26)     CCCCTTGCACCCTCTCAACAT
27)     CCACCTGCCAGTGGATGTTGATA
28)     CTGGGGTGCACCCTCCCAGTAT
29)     CCCCACTGCACCCCTGGCAT
30)     CCCCCCCGCACCCCTCCAGCAT
31)     TCCCCTCGCACCCTCCCAGTAT
32)     CCCCCTGCATCCCAGGCTCC
AGGCCTCCCGCCCACCTTGCGGTCCCCGCCCTGGCGTCTAGGTGGCACTAGAATCCCGTGCTGACTCCTCCCGCTGGGAGCCGCCCTCGCCTGC
CCATGGTTGTCCAGCTCCGTCCCTCTATATGCTGAGCCCAACCGGCTGCACAGTTTTCAGGGGTCAGTTCCTCCAAGTAAAGGGGCTGTGGCT
TCCCTGGAGCTGCAGACTTGTCATTGCTTTTTTCTTTCTGTCTTCTACTTCCTATGGTTCTTGGCCTCCTCTTGGGGAGAGGTAGAGGAGCCCT
CTCCTCTCCGCCTCAGGGACAACCCAAAGCAAGTACTGCATGTGCTCTTTTAAAAGTTTTAAATAATTTTAGCAAAAGGATATTAACATTAAAT
CAATTTTTAAACTTTTTGAAAAAAGTATCAAAACAACATGCACGTGGTTCAAAACAGTAGGCTCCTCTTGGGCCCTTTCAGATAATTCAAACTG
TCACTCAGGCTGGAGTGCCACGGCTCACTGCAGCTTCGACCTCCCGGGCTCAGCTGATCCTCCCACCTCAGCCTCCTGAGTAGTAGCTGGGACT
ACAGCGCGGGCAACCACACCCAGCTAATTAAAAAAAAAATTCTAAGAGATGGGGTCTTGCTGTGTTGCCCAGGCTGGTCTTGAACTCCTGGGCTC
AAGCGATCCTCTCGCCTCAGCCTCCCAAAGCACTGTGCTCTGTTTTGACGCTGCTTTGAACTGTAGCTGGTTAAAAAATGAGAACCAGTTCTTC
ATTCCTTCATTCTGCAAGTATTTATTGTGAGGCTCTGGGGACGGAGAGGAATTACACAAGGGCCTCTACGCCGAGCTCACATCCCAGCGGGGCA
GTCAGAGAAACCCGTGGGTACCACGTGTTGTTACGTCCCAGGAAGAAAGGGAGAGCAGGTTTCAGGATGGGGCCATGGGGAGGTGTCAGGGGTC
AAGAGAGATGGCTGGGAGGAAGCTCCACAAGCAGAACCCTACACGCGGTCCAAGAAGGGTGCCGGCAGGGGAGGCGCATACCCGCCCCTTTGTG
TAGCCTCCCCCTTCCCTCAAAGTTAAAGAGCAGGAAAGTCAGGATTCCTCGCCGGCCCTGCCCTGCCGAATGCTCCGCGCTCCGCTCCTGCCTG
CGAGCTTGTGTGTATCGGGGGTCCCTCCCCTCCCCGCGCTGGGATCCGGCGCGCACCCCGCCCCGCAGCGCGGCCCCTCCCGGGCGAGCGCACC
CCCATCCATTCAGCTGGAGCGCCGAGCCGCGGCCGCGGGGAAGCATTAAGTTTACTCGCCTCAAAGTGACGCAAAAATTCTTCAAGAGCTCTTT
GGCGGCGGCTATCTAAAGATCGGACCATGTGAGGGACCGCGGGTACAAATACCGCCGCGCCGGCGCCCCTCCGCACAGCCAGCGCCGCCCGGTG
CCTCGAGGGTGCGAGGCCTGACCACCTCCCCAGCCCGGGACCAGCCTCCGCCTGGCAGTCCGGCAGGTGGGTCCGCTTTTCCTCTCCGCCTCGA
ACCCACGCTTCTTCC +129 kb
```

Figure S2| Consensus sequence of the marmoset *SLC6A4* promoter region. A 2.40 kb fragment from -2.3kb and the first exon was cloned, sequenced and aligned and a consensus sequence determined, accession number HG515029. The transcription start site that we determined experimentally is highlighted in black (+1A). Exon 1 is shaded yellow based on *genebuild* from the Ensembl database. A region containing 32 tandem-repeats was identified; each individual repeat is numbered above. The 3rd, 4th and 23rd repeats (red bold) contain the marmoset sequence polymorphisms (in this case AC/C/G). Primers used for cloning and genotyping are underlined (see **Table S2**).

Figure S3:

```
mac       AAAAAAAAAAGAACGAGAAGTGGAACGTGGGAGGCAGCAGACAACTGTGTTCATCTGAAA
marm      AAACATGTTCAC---TTAAGTGGAACATTGGAGGCAGCAAACAACCGTGTTCATCTGAAA
hum       AAACATGCTCATTTAAGAAGTGGAACGTGGGAGGCAGCAGACAACTGTGTTCATCTGAAA
          ***.*:. :..    : *********.* **********.***** **************

mac       GGAGGAGCCCCCACCCCCATGCAGGCGCTGCCCCTGGGGATGAAATTCCCAAGCTTGTTG
marm      GGAGGAGCCCCCACCCCCATGCAGTCACTGCCCCCAT-AATAAAATTCCCAAGCTTGTTG
hum       GGAGGAGGCCCCACTCCCGTGCAGGCGCTGCCCCTGGGGGTGAAATTCCCAAGCTTGTTG
          ******* ****** ***.***** *.******* .  ..*.******************

mac       GGGAATCCCCCGCCTGGCGTTGCCGCTCTGAATACCAGCAC---CTAA-CCCCCTAATGT
marm      GGGAATCC-CCACTAGGCATTGCTACTCTGAATGCCAGCAGCACCTAACCCTCCTAACGT
hum       GGGATTCTCCCGCCTGGCGTTGCCGCTCTGAATGCCAGCAC---CTAA-CC-CCTAATGT
          ****:**  **.* :***.**** .********.******    **** ** ***** **

mac       CCCTGCTGCAGCCCTCCCAGCATCTCCC--------------------------------
marm      CCCTGCTGCACTCTTCCCTTCACCCCCCACCCCCCGCACCCTCCCAGCATACCCCCTGCA
hum       CCCTACTGCAGCCCTCCCAGCATCCCC---------------------------------
          ****.*****  * ****: ** * **---------------------------------
                 First repeat
mac       -------------------TGTACCCCTCCTAGGATCTCCCCTGCACCCCCCATTATCCT
marm      ACTCTCCCATCATTCCCTCTGCACCCCTCCCAGCATTCCCCCTGCACCCTCCCAA--CAT
hum       ------------------------------------------------------------

mac       CCCTAC------------ACCCCCCAGCATCCCCCCTGCAGCCTCCCAGCATCTCCCCTG
marm      CCCTCCTCCCCTCTGCACCCCTCCCAGCATTCGCCCTGCACCCTCCCAGCATCCCCCTTC
hum       ----------------------------------CCTGCAACCTCCCAGCAACTC-----
          ----------------------------------****** **********:* *-----

mac       C------------ACCCCCCAGCATCC----------CCCCTGCACCCCTCCCAGGATCT
marm      ACCCCTCCCTTCGTCCCCCCTGCATCCTCCCAGCATCCCCCTGCACCCCTCCAATCATCC
hum       ---------------------------------------CCTGTACCCCTCCTAGGATCG
          ---------------------------------------**** ******** *  ***-

mac       CCCTTTCATCCCCATTATCTCCCCTGCACCCCTCGCAGTATCCCCCCGC--ACCTC----
marm      CCCCTGCATCCTCCC--ACCCCTCTGTACCCCTCCCAGCATCCCCCATGAATCCTCCCAA
hum       CTCCTGCATCCCCCA---------------------------------------------
          * * * ***** *.----------------------------------------------

mac       ----CATTATCCCCCCTGCACCCCTTGCGGCATC--------------------------
marm      AAACCTCCCTCCCCCCGGAACCCCTCCCAGCATTCCCCTGCACCCCTCCAGCATCTCCCT
hum       -----TTATCCCCCCCTTCACCCCTCGCGGCATC--------------------------
               :    ******  .******  *.****---------------------------

mac       -------------CCCCCTACACCC-CCCAGTATTCCCCCTGCAGCACCCCCCAGCATCT
marm      TGCCCCAGACGTCGCCCCTGCACCCCTCCAGCATTCCCCTTG---TGCCCCCCAGAATCT
hum       -------------CCCCCTGCACCC--CCAGCATCCCCCCTGCAGCCC-CCCCAGCATCT
                        *****.*****  **** ** **** **     * ******.****

mac       CCCCCGCA--CCGCCCAGCATCCCCCCTGCAGC-CCTTCCAGCAACC-CCTGCATCCCTC
marm      CCCTTATACCTCTCCCAGCATCCCCCCCGTAACCCCTCCCAGCATCCCCCTGCACCCCTC
hum       CCCCTGCA--CC-CCCAGCATCCCCCCTGCAGC-CCTTCCAGCATCCCCCTGCACCTCTC
          ***  . *   * ************** * *.* *** ******:** ****** * ***

mac       CCAGGATCTCCCC-------------------TG-----------CAACCC-CAT-----
marm      C-AGCATCTCCCTTGCCCCTAGACGTCACCCCTGCACCCGTCCAGCATTCCCCTTGTGCC
hum       CCAGGATCTCCCC-------------------TG-----------CAACCCCCAT-----
          * ** *******                    **           **: ** *:*

mac       -----TATCCCCCCTGCACCCCTCGCGGCATCCC-CC-----------------------
marm      CCCCAGATCTCCCCTATACCTCTCCCAGCATCCCCTTGCACCCTCTCAACATCCACCTGC
hum       -----TATCCCCCCTGCACCCCTCGCAGTATCCCCCC-----------------------
                *** *****. *** *** *.* *****----------

mac       ---------------------TACACCC-CCCAACATACCCTCTGCACCCCCCGGAATCC
marm      CAGTGGATGTTGATACTGGGGTGCACCCTCCCAGTATCCC-CACTGCACCCCTGGCATCC
hum       ---------------------TGCACCC-CCCAGCATCCCCCCATGCACCCCCGGCATCC
                               *.***** ****. **.**  .   ..**** **.****

mac       CCCCTGCACCCCTCCAGCATTCCCCTTGCACCCTCCCAGTATCCCCCTGCATCCCGGGCT
marm      CCCCCGCACCCCTCCAGCATTCCCCTCGCACCCTCCCAGTATCCCCCTGCATCCCAGGCT
hum       CCCCTGCACCCCTCCAGCATTCTCCTTGCACCCTACCAGTATTCCCCCGCATCCCGGCCT
          **** ***************** *** *******.******* **** *******.* **
                                                    Last repeat
mac       CCAAGCCTCCCGCCCACCTTGCGGTCCCCGCCCTGGCGTCTAGGTGGCACCAGAATCCCG
marm      CCAAGCCTCCCGCCCACCTTGCGGTCCCCGCCCTGGCGTCTAGGTGGCACTAGAATCCCG
hum       CCAAGCCTCCCGCCCACCTTGCGGTCCCCGCCCTGGCGTCTAGGTGGCACCAGAATCCCG
          ************************************************** *********

mac       CGCGGACTCCTCCCGCTGGGTGCCGCCCTCGCTTGCCCATGGTTGTCCAGCTCAGTCCCC
marm      TGCTGACTCCTCCCGCTGGGAGCCGCCCTCGCCTGCCCATGGTTGTCCAGCTCCGTCCC-
hum       CGCGGACTCCACCCGCTGGGAGCTGCCCTCGCTTGCCCGTGGTTGTCCAGCTCAGTCC--
           ** ******:*********:** ******** *****.*************.****

mac       CTCTAGACGCTCAGCCCAACCGGCCGCACAGTTTTCAGGGGTCAGTTCCTCCAAGTACAA
marm      -TCTATATGCTGAGCCCAACCGGCTGCACAGTTTTCAGGGGTCAGTTCCTCCAAGTAAAA
hum       CTCTAGACGCTCAGCCCAACCGGCCGCACAGTTTTCAGGGGTCAGTTCCTCCAAGTACAA
           **** * *** ************ ********************************.**
```

Figure S3| *SLC6A4* repeat region alignment using the online tool Clustal Omega (http://www.ebi.ac.uk/Tools/msa/clustalo/). The human and macaque genomic regions were downloaded from the Ensembl database (www.ensembl.org). The sequences shown span the full *SLC6A4* repeat region flanked by ~200bp upstream and downstream sequence. The first and last repeats are highlighted in red. The repeat region was aligned manually in a second alignment shown in **Fig. S4.** “mac”: rhesus macaque; “marm”: common marmoset; “hum”: human.

Figure S4

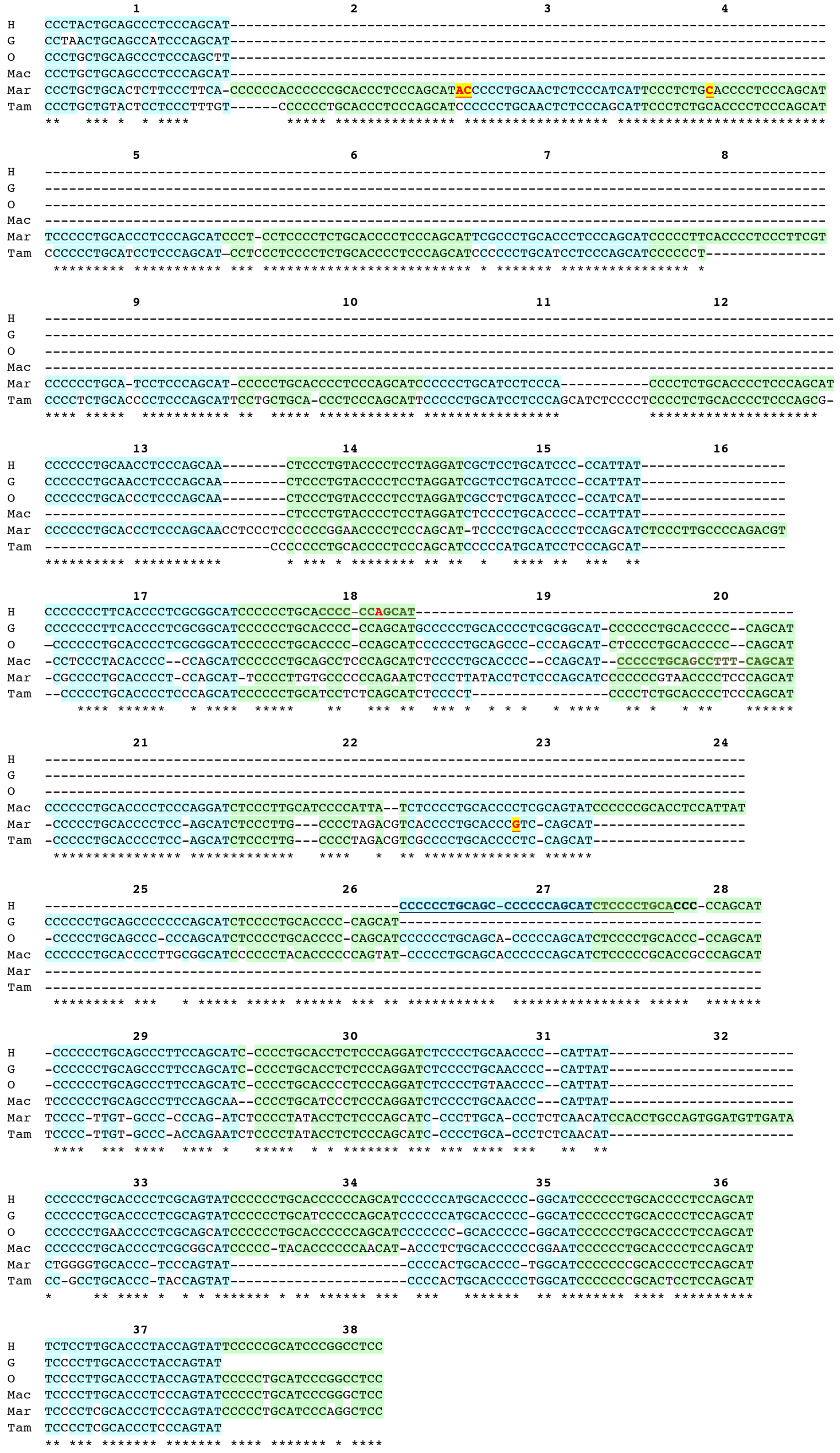

Figure S4| Multiple sequence alignment of repeat blocks in the human and non-human primate *SLC6A4* upstream repeat region. Repeats were manually aligned and alternately highlighted in blue and green. Bases conserved between all species are marked with an asterisk (*). Human (H) and rhesus macaque (Mac) insertion/deletions are underlined and highlighted in bold. Importantly, the marmoset (Mar)

polymorphic sequences in the 3rd, 4th and 23rd repeats (AC/C/G in this case) and the human G/A single nucleotide polymorphism in the 6th human repeat (A in this case, repeat no. 18) are underlined and highlighted in red. 26-repeat tamarin (Tam), 21-repeat orang-utan (O) and 19-repeat gorilla (G) examples are shown. Whilst some repeats are shared by all species (repeat no. 1, 14-15, 17-18, 29-31, 33, 35-37) some others are exclusive to one single species (repeats no. 8, 16, 32 for marmoset and 24 for macaque). Gorilla sequence is incomplete in the 3' end (repeat no.38). See Methods for accession numbers.

Figure S5

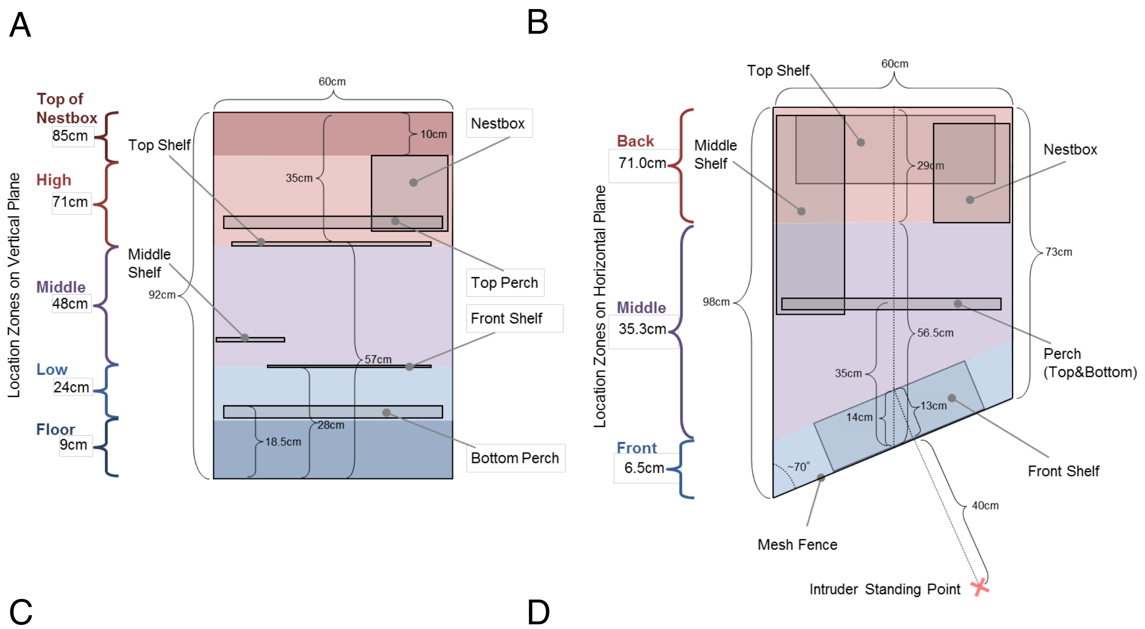

C

Hypotenuse (cm) from Front & Floor		Horizontal Zone and Middle Point (cm): Back	Middle	Front
		71.0	35.3	6.5
Top of Nestbox	85.0	110.75	91.92	85.25
High	71.0	100.41	79.16	71.30
Middle	48.0	85.70	59.41	48.44
Low	24.0	74.95	42.44	24.86
Floor	9.0	71.57	36.14	11.10

Vertical Zone and Middle Point (cm)

D

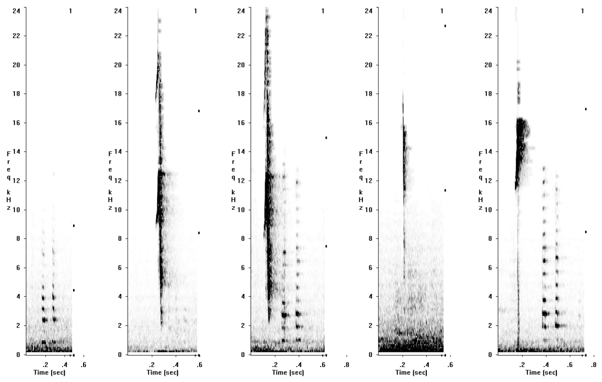

Figure S5 | Average distance and marmoset calls during Human Intruder Test. The test quadrant was divided into fifteen locations (*n*). **A** and **B**) Schemes of the home cage test quadrant indicating the positions corresponding to more anxiety shaded in red and less anxiety, in blue: **A**) front view: top of the nest box, high, middle, low and floor and **B**) top view: back, middle and front. **C**) Table showing the hypotenuses from the middle point of each location *n* to the front-floor location, nearest point to the human intruder. The average distance was calculated using the proportion of time spent on each location *n*, summed across all 15 locations. **D**) Examples of the five types of calls recorded, with their typical bandwidth and pattern of frequency graphically represented against time in sonograms (using the program Syrinx).

Figure S6

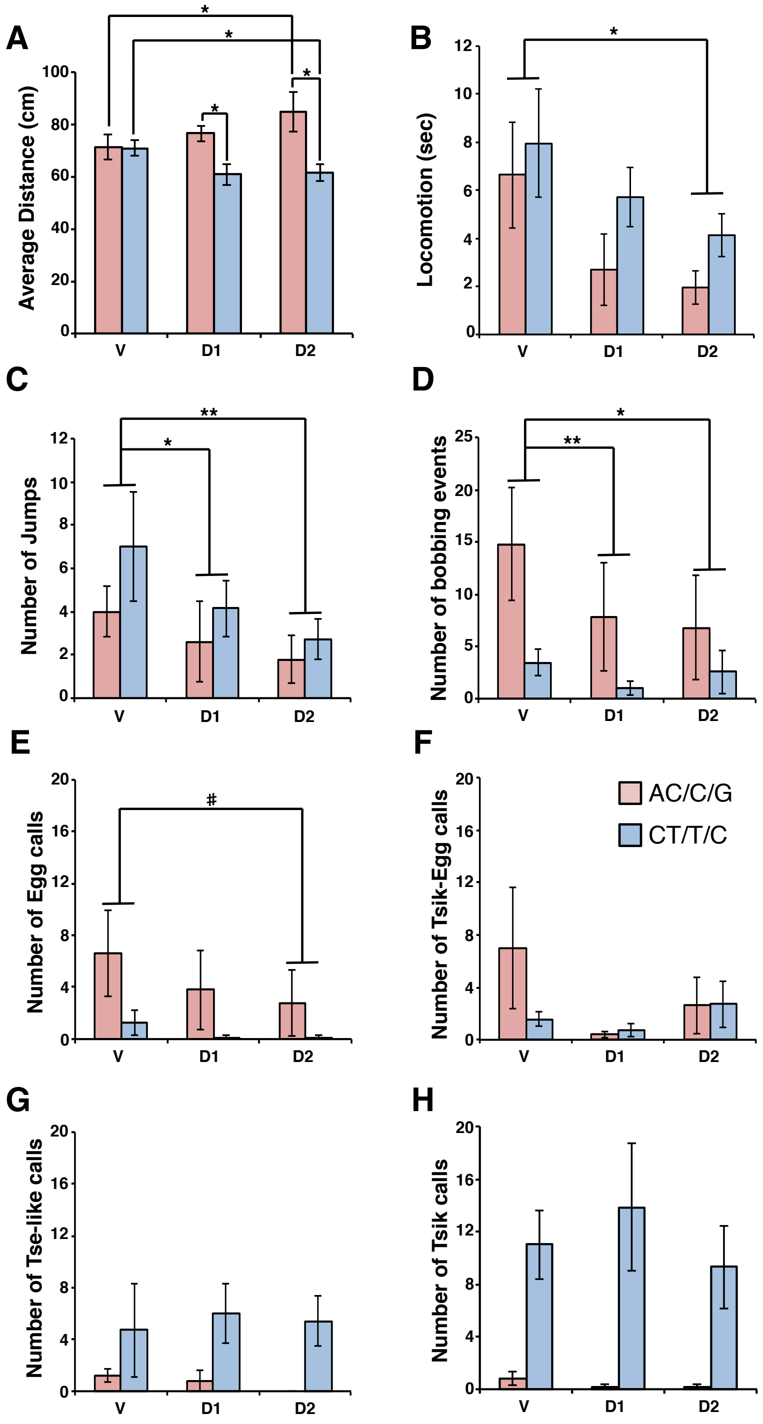

Figure S6 | Anxiety response to a selective serotonin reuptake inhibitor, citalopram. Human Intruder Test (HIT) was used to assess anxiety levels in response to vehicle (V) and to a single dose of 2.5 mg/kg (D1) or 10 mg/kg (D2) citalopram, 25 minutes prior the *intruder phase*. All variables were scored and compared between genotypes using Repeated Measures ANOVA, with within factor 'treatment' and between factors 'genotype'. **A)** *Average distance.* Treatment x genotype interaction $F_{(2)}$=4.214 p=.030; followed by LSD pairwise comparisons. D1: AC/C/G vs CT/T/C p=.017; D2:

AC/C/G vs CT/T/C p=.012; AC/C/G: V vs D2 p=.012 and CT/T/C: V vs D2 p=.031. **B**) *Locomotion*. Main effect of treatment Greenhouse-Geisser $F_{(1.344)}$=6.579 p=.017; followed by LSD pairwise comparisons: V vs D2 p=.01. **C**) *Number of jumps* (square root transformed). Main effect of treatment $F_{(2)}$=9.997 p=.001; followed by LSD pairwise comparisons: V vs D1 p=.040 and V vs D2 p=.001. **D**) *Head and body bobbing* (square transformed). Main effect of treatment $F_{(2)}$=8.525 p=.002; followed by LSD pairwise comparisons: V vs D1 p=.007 and V vs D2 p=.016. **E**) *Number Egg calls*. Main effect of treatment Greenhouse-Geisser $F_{(1.302)}$=3.621 p=0.071. **F**) *Tsik-Egg* **G**) *Tse-like calls* and **H**) *Number of Tsik*, NS. Mean±s.e.m., * p<.05, ** p<.005, # p<.08 (trend).

Video S1: High anxious marmoset behavior. Representative example of the emotional response during the *intruder phase* of the Human Intruder Test for an AC/C/G homozygous marmoset. Marmosets maintain a large distance between themselves and the unfamiliar human, staying on top of the nest box. They also show reduced locomotion and perform high numbers of head and body bobbing and alarm Egg calls.

Video S2: Low anxious marmoset behavior. Representative example of the emotional response during the *intruder phase* of the Human Intruder Test for a CT/T/C homozygous marmoset. Marmosets approach the unfamiliar human, show increased locomotion and perform high numbers of mobbing Tsik calls.

References

Barros M (2002). Reactions to Potential Predators in Captive-Born Marmosets (Callithrix penicillata). *Int J Primatol* **23**: 443–454.

Bezerra BM, Souto A (2008). *Structure and Usage of the Vocal Repertoire of Callithrix jacchus*. *Int J Primatol* **29**: .

Cross N, Rogers LJ (2006). Mobbing vocalizations as a coping response in the common marmoset. *Horm Behav* **49**: 237–45.

Shiba Y, Kim C, Santangelo AM, Roberts AC (2015). Lesions of either anterior orbitofrontal cortex or ventrolateral prefrontal cortex in marmoset monkeys heighten innate fear and attenuate active coping behaviors to predator threat. *Front Syst Neurosci* **8**: 250.
