# Supplementary figures and images for: Novel Primate Model of Serotonin Transporter Genetic Polymorphisms Associated with Gene Expression, Anxiety and Sensitivity to Antidepressants
Source: Neuropsychopharmacology. 2016 Apr 20;41(9):2366–76. doi: 10.1038/npp.2016.41 (PMC4946067; doi:10.1038/npp.2016.41)

Figure S1

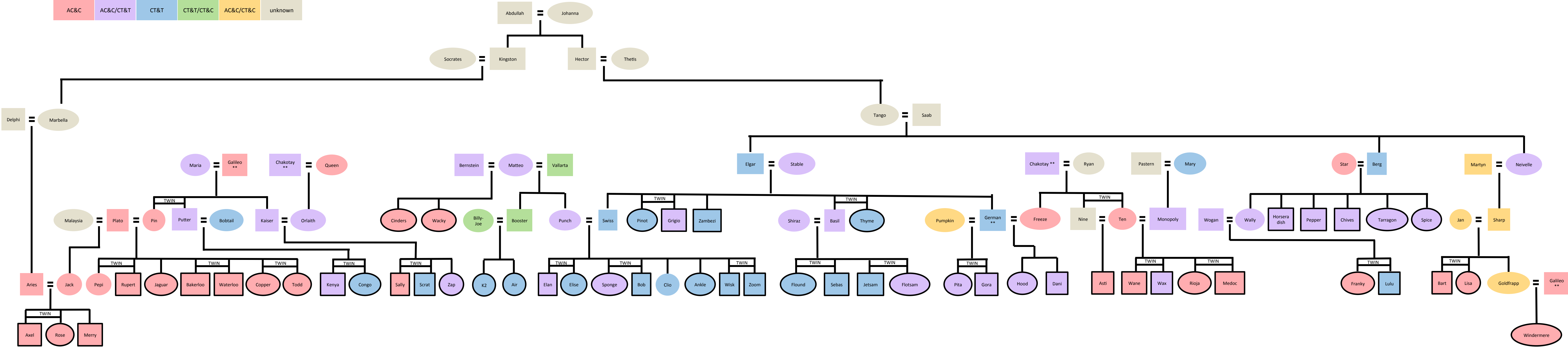

Supplement: Supplementary Figure S1 [file npp201641x2.pdf]
